# Molecular subclass of uterine fibroids predicts tumor shrinkage in response to ulipristal acetate

**DOI:** 10.1093/hmg/ddac217

**Published:** 2022-09-01

**Authors:** Åsa Kolterud, Niko Välimäki, Heli Kuisma, Joonatan Patomo, Sini T Ilves, Netta Mäkinen, Jaana Kaukomaa, Kimmo Palin, Eevi Kaasinen, Auli Karhu, Annukka Pasanen, Ralf Bützow, Oskari Heikinheimo, Helena Kopp Kallner, Lauri A Aaltonen

**Affiliations:** Department of Biosciences and Nutrition, Karolinska Institutet, 141 83 Huddinge, Sweden; Department of Medical and Clinical Genetics, University of Helsinki; Applied Tumor Genomics Research Program, Research Programs Unit, University of Helsinki, 00290 Helsinki, Finland; Department of Medical and Clinical Genetics, University of Helsinki; Applied Tumor Genomics Research Program, Research Programs Unit, University of Helsinki, 00290 Helsinki, Finland; Department of Medical and Clinical Genetics, University of Helsinki; Applied Tumor Genomics Research Program, Research Programs Unit, University of Helsinki, 00290 Helsinki, Finland; Department of Medical and Clinical Genetics, University of Helsinki; Applied Tumor Genomics Research Program, Research Programs Unit, University of Helsinki, 00290 Helsinki, Finland; Department of Medical and Clinical Genetics, University of Helsinki; Applied Tumor Genomics Research Program, Research Programs Unit, University of Helsinki, 00290 Helsinki, Finland; Department of Medical and Clinical Genetics, University of Helsinki; Applied Tumor Genomics Research Program, Research Programs Unit, University of Helsinki, 00290 Helsinki, Finland; Department of Medical and Clinical Genetics, University of Helsinki; Applied Tumor Genomics Research Program, Research Programs Unit, University of Helsinki, 00290 Helsinki, Finland; iCAN Digital Precision Cancer Medicine Flagship, University of Helsinki, 00290 Helsinki, Finland; Department of Medical and Clinical Genetics, University of Helsinki; Applied Tumor Genomics Research Program, Research Programs Unit, University of Helsinki, 00290 Helsinki, Finland; Department of Medical and Clinical Genetics, University of Helsinki; Applied Tumor Genomics Research Program, Research Programs Unit, University of Helsinki, 00290 Helsinki, Finland; Department of Pathology, University of Helsinki and HUSLAB, Helsinki University Hospital, 00290 Helsinki, Finland; Department of Pathology, University of Helsinki and HUSLAB, Helsinki University Hospital, 00290 Helsinki, Finland; Department of Obstetrics and Gynecology, University of Helsinki and Helsinki University Hospital, 00029 HUS Helsinki, Finland; Department of Obstetrics and Gynecology, University of Helsinki and Helsinki University Hospital, 00029 HUS Helsinki, Finland; Department of Clinical Sciences, Danderyd Hospital, Karolinska Institutet, 171 77 Stockholm, Sweden; Department of Obstetrics and Gynecology, Danderyd Hospital, 182 88 Stockholm, Sweden; Department of Biosciences and Nutrition, Karolinska Institutet, 141 83 Huddinge, Sweden; Department of Medical and Clinical Genetics, University of Helsinki; Applied Tumor Genomics Research Program, Research Programs Unit, University of Helsinki, 00290 Helsinki, Finland; iCAN Digital Precision Cancer Medicine Flagship, University of Helsinki, 00290 Helsinki, Finland

## Abstract

Precision medicine carries great potential for management of all tumor types. The aim of this retrospective study was to investigate if the two most common genetically distinct uterine fibroid subclasses, driven by aberrations in *MED12* and *HMGA2* genes, respectively, influence response to treatment with the progesterone receptor modulator ulipristal acetate. Changes in diameter and mutation status were derived for 101 uterine fibroids surgically removed after ulipristal acetate treatment. A significant difference in treatment response between the two major subclasses was detected. *MED12* mutant fibroids had 4.4 times higher odds of shrinking in response to ulipristal acetate treatment as compared to *HMGA2* driven fibroids (95% confidence interval 1.37–13.9; *P* = 0.013), and in a multivariate analysis molecular subclassification was an independent predictive factor. Compatible with this finding, gene expression and DNA methylation analyses revealed subclass specific differences in progesterone receptor signaling. The work provides a proof-of-principle that uterine fibroid treatment response is influenced by molecular subclass and that the genetic subclasses should be taken into account when evaluating current and future uterine fibroid therapies.

## Introduction

Uterine fibroids (UFs), also known as leiomyomas, are benign clonal tumors that occur in up to 70% of women of childbearing age (1). In approximately 25% of these women they cause symptoms such as heavy menstrual bleeding and anemia, abdominal pain and discomfort, pregnancy complications and infertility (2), affecting quality of life and resulting in significant health care costs.

UFs arise from the smooth muscle wall (myometrium) and are characterized by excessive deposition of extracellular matrix (ECM). UF growth is estrogen and progesterone dependent and symptoms often attenuate at menopause (2,3). UFs are histologically and symptomatically heterogeneous, and can grow at strikingly different rates. Occurrence of multiple synchronous tumors is common.

High-throughput analyses by us and others have revealed at least four molecularly distinct UF subclasses, harboring mutually exclusive driver gene mutations (4,5). The majority of UFs (70%) carry mutations in the *mediator complex subunit 12* (*MED12*) (6). The second most common driver gene aberration, seen in approximately 10% of UFs, is translocation in the *high mobility group AT-hook 2* (*HMGA2*) gene, resulting in upregulated *HMGA2* expression (7,8). Hereafter, these subclasses are referred to as MED12 and HMGA2. Biallelic inactivation of the tricarboxylic acid cycle gene fumarate hydratase is seen in 1–2% (9), and SRCAP complex gene mutations in 2% of UFs (5).

The mutually exclusive driver gene changes result in unique gene expression profiles (5) suggesting that fibroids can arise through different pathogenic mechanisms. The subclasses have also been associated with distinct clinical and pathological characteristics. HMGA2 fibroids tend to be larger, with an increased growth rate and higher vessel density (10,11). MED12 fibroids are typically smaller and subserosal (12). Despite the recent advancements robustly deciphering UFs intrinsic biological diversity, the clinical relevance of the genetic UF subclasses has not been established.

Only surgical removal by hysterectomy or myomectomy is curative. Two types of volume-reducing drug therapies are currently approved for UF treatment in Europe; gonadotropin releasing hormone (GnRH) analogs that inhibit pituitary gonadotropin secretion, and the selective progesterone receptor modulator ulipristal acetate (UPA). Whereas fibroids rapidly regrow after GnRH treatment, UPA-induced volume reduction may persist for months following treatment cessation (13). Nevertheless, as many as one-third of treated fibroids fail to shrink in response to treatment, and some continue growth during treatment (14–16). It is currently not possible to predict which fibroids will shrink in response to treatment.

Rational design of new management options is needed to efficiently reduce the burden UFs impose to women’s health. We hypothesize that taking the genetic subclassification of UFs into account could significantly enhance such efforts. To test this hypothesis, we examined whether variation in UPA treatment response correlates with the two most common UF subclasses, MED12 and HMGA2.

## Results

### Sample collection and determination of treatment response

**Figure 1:**
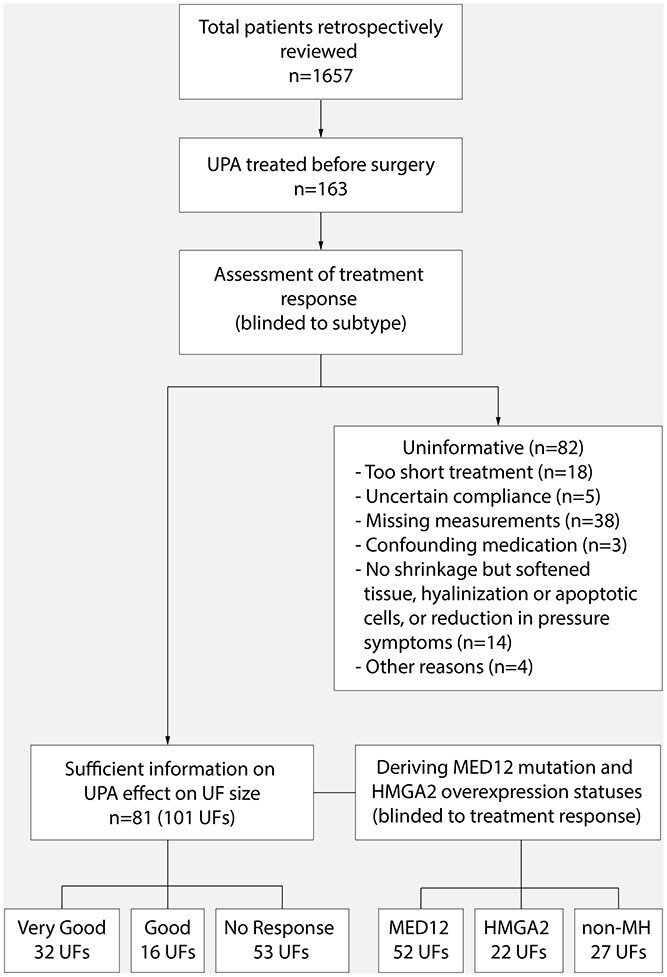
Study flowchart. Overview of the study workflow, patients reviewed and fibroids included. UPA: ulipristal acetate; UF: uterine fibroid; non-MH: wild type for *MED12* and *HMGA2.*

In total, we harvested 101 tumors from 81 women who had received UPA treatment for UF related symptoms prior to surgery, and for which treatment response defined as fibroid shrinkage, could be robustly retrieved from the respective patient records (Fig. 1, Supplementary Material, Table S1; summary in Supplementary Material, Table S2). Whenever available, the following information was derived from the patient records: duration of UPA treatment, measurements of fibroid size before and after treatment (largest diameter measured by ultrasound or in some cases magnetic resonance imaging (MRI)), written assessments of treatment response, and pathology reports. UFs were considered very good responders (VGR) when records showed tumor size reduction of at least one centimeter in diameter or described shrinkage in unambiguous terms such as ‘excellent’ or ‘large’. Fibroids with lesser size reduction—shrinkage described in terms such as ‘slight’, ‘some’ but not with vague expressions such as ‘maybe’—were considered good responders (GR). UFs remaining stable or increasing in size and for which the records did not indicate reduced pressure symptoms, tissue softening, hyalinization or apoptotic cells, were considered non-responders (NR).

Of the 101 fibroids, 48 were categorized as VGR/GR, and 53 as NR to UPA treatment. The proportion of VGR/GR UFs in our cohort (48%) is somewhat smaller than the around 60% (up to 78% after several treatment courses) reported earlier (16,17). This is likely due to the fact that the UFs that responded best to treatment did not need to be surgically removed and thus were not available for genetic subclassification.

The baseline characteristics, duration of UPA treatment, pre-treatment fibroid size, total number of fibroids, tumor location, parity, age at surgery and type of surgery for each treatment response category are outlined in Table 1 (details in Supplementary Material, Table S1).

**Table 1:** Treatment response

	NR	GR	VGR
Number of patients	45	10	26
Duration of UPA treatment (months)	3 (3–6)	3 (3–6)	3 (3–6)
Age at surgery (years)	45 (42–50)	45 (42–49)	46 (44–49)
Operation			
Hysterectomy	39 (86.7%)	8 (80.0%)	25 (96.2%)
Myomectomy	6 (13.3%)	2 (20.0%)	1 (3.8%)
Nulliparous	14 (31.1%)	3 (30.0%)	5 (19.2%)
Total number of UFs per patient	1 (1–2)	1.5 (1–2)	3 (2–4)
Number of UFs in this study	53	16	32
UF diameter at start (mm)	55 (42–74)	60 (38–77)	56 (44–71)
Location			
Intramural	29 (74.4%)	4 (40.0%)	22 (78.6%)
Submucous	7 (17.9%)	4 (40.0%)	4 (14.3%)
Subserous	3 (7.7%)	2 (20.0%)	2 (7.1%)

Patient characteristics are summarized according to the treatment response of the largest fibroid at the onset of treatment. Data are shown either as a median and interquartile range or as a number and percentage. Location information was available for 77 fibroids. UFs: uterine fibroids, UPA: ulipristal acetate, NR: non-responders, GR: good responders, VGR: very good responders.

### Genetic subclassification of UFs

Altogether 52 out of the 101 tumors carried *MED12* mutations (Finland *n* = 34; 61%, Sweden *n* = 18; 40%), the majority of these being single base substitutions in codon 44 as expected (18). We next screened all samples lacking *MED12* mutations for *HMGA2* overexpression and identified 22 *HMGA2* overexpressing samples (Finland *n* = 10; 18%, Sweden *n* = 12; 27%). The tumors not carrying *MED12* mutations or overexpressing *HMGA2* were categorized as non-MED12-HMGA2 (non-MH; *n* = 27; 27%). The frequency of MED12 UFs (51%) was lower than expected (70%) (4), presumably due to small tumors being underrepresented in the Swedish set (Supplementary Material, Fig. S1). MED12 UFs were, as expected, somewhat smaller at the start of treatment (diameter median 50 mm; IQR 40–70 mm) than HMGA2 (60 mm; 52–75 mm) and non-MH UFs (60 mm; 47–77 mm) (12) (Supplementary Material, Fig. S1).

### Correlation between genetic UF subclass and response to UPA treatment

**Figure 2:**
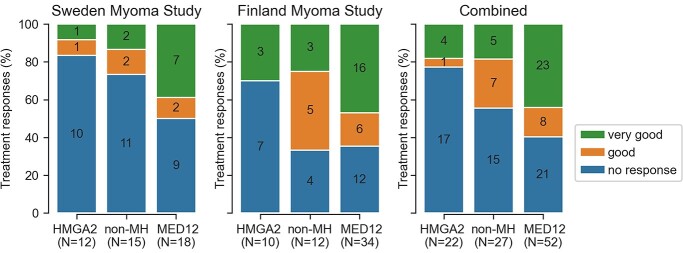
Treatment responses stratified by subclass. Data shown as percentages (stacked bar plot) and as exact numbers of tumors (*N*). From left to right, Sweden and Finland Myoma Study, and the two combined. Green: very good treatment response; orange: good response; blue: no response; non-MH: wild type for *MED12* and *HMGA2*.

A total of 31/52 MED12 UFs (60%) fell into VGR/GR category, as compared to 5/22 HMGA2 UFs (23%) and 12/27 of the non-MH UFs (44%). We found a statistically significant difference in treatment response between the two major UF subclasses, with MED12 UFs having 4.4 times higher odds to UPA induced size reduction compared to HMGA2 UFs (95% confidence interval (CI) 1.37–13.9; *P* = 0.013; VGR/GR versus NR; Fig. 2). The majority of this effect was due to VGR UFs, where MED12 UFs had 4.7 times higher odds compared to HMGA2 (95% CI 1.30–16.9; *P* = 0.018; VGR versus NR). Also, MED12 UFs seemed to have a better mean response than non-MH UFs, but the effect size was non-significant (OR = 1.6; 95% CI 0.6–4.4; *P* = 0.35; VGR/GR versus NR; Fig. 2), and the majority of this difference was due to VGR UFs (OR = 3.5; 95% CI 1.03–12.2; *P* = 0.045; VGR versus NR). The above-mentioned differences were also reflected in our measurements of tumor size changes from start to end of treatment (Supplementary Material, Fig. S2).

None of the background variables explained the observed difference in treatment response. Adjusting for patient age at surgery (years), tumor diameter at start of treatment (mm), parity (nulliparous or parous), total number of fibroids (one or multiple) and cohort (Sweden or Finland Myoma Study) resulted in a similar effect size, MED12 UFs having 4.6 times higher odds of treatment response compared to HMGA2 UFs (95% CI 1.33–16.1; *P* = 0.016; Supplementary Material, Fig. S3). Adjusting for these background variables, MED12 UFs displayed also somewhat better mean response than non-MH UFs, but this difference was not significant (OR = 1.2; 95% CI 0.4–3.7; *P* = 0.75).

Histopathological characteristics of the UPA-treated samples were also evaluated to determine if necrosis, edema and hemorrhage would explain, in part, the observed differences in treatment response. Altogether 53 UFs were available for scoring (Supplementary Material, Table S3). Necrosis and hemorrhage positivity were rare and no statistical differences could be derived: four tumors were positive for necrosis (2 NR MED12; 1 GR non-MH; 1 VGR HMGA2) and one tumor was positive for hemorrhage (VGR non-MH). Edema positivity was overall more common and could be stratified with respect to treatment response and the two major UF subclasses: among non-responding tumors, HMGA2 had elevated edema positivity (5/7 or 70%) compared to MED12 (4/10 or 40%), but the difference was not significant (OR = 3.8; *P* = 0.3; two-sided Fisher’s exact test). Among VGR/GR tumors, HMGA2 (1/3) and MED12 (7/21) had no difference in edema positivity (OR = 1.0; *P* = 1.0). Finally, a complementary set of untreated tumors was evaluated to determine a baseline for edema positivity in HMGA2 and MED12 tumors: untreated HMGA2 (16/33 or 48%) had more edema compared to untreated MED12 (15/34 or 44%), similar to what has been proposed previously (19). No significant differences between untreated-HMGA2 and untreated-MED12 (OR = 1.19; *P* = 0.8), or between untreated-HMGA2 and NR-HMGA2 (OR = 2.7; *P* = 0.4), were found.

### Evidence for differences in progesterone receptor signaling in MED12 and HMGA2 UFs

**Figure 3:**
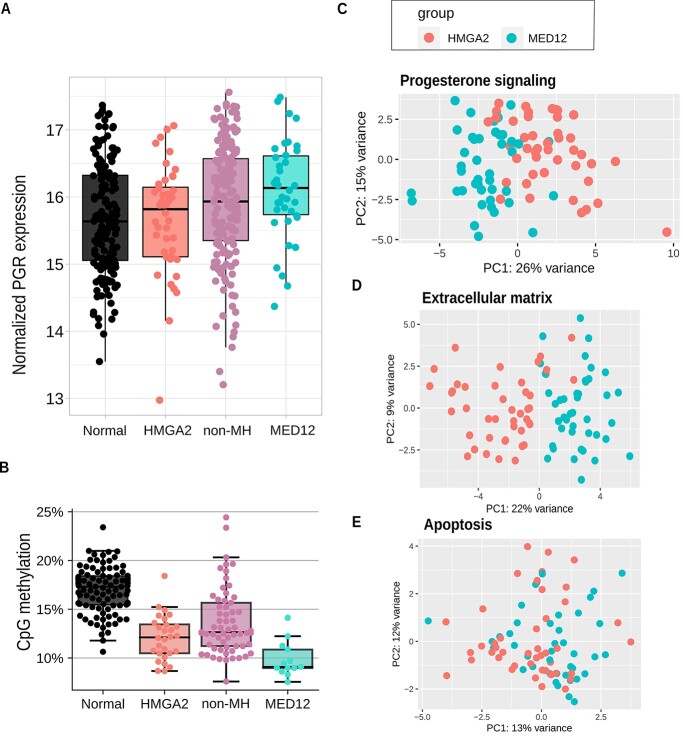
Signaling differences in MED12 and HMGA2 UF samples. (**A**) Progesterone receptor (PGR) expression in normal myometrium (*n* = 162), HMGA2 UFs (*n* = 44), MED12 UFs (*n* = 42) and UFs wild type for *HMGA2* and *MED12* (non-MH, *n* = 175). *Y*-axis shows log-scaled estimates of gene expression. (**B**) Average CpG methylation at PGR binding sites in normal myometrium (*n* = 96), HMGA2 UFs (*n* = 28), MED12 UFs (*n* = 13) and UFs wild type for *HMGA2* and *MED12* (non-MH, *n* = 61). (**C**) PCA of 48 progesterone-related genes, (**D**) 84 ECM-related genes and (**E**) 90 apoptosis-related genes. HMGA2 UFs are represented by red dots and MED12 UFs by blue dots.

To obtain mechanistic insight into the observed difference in treatment sensitivity between the two subclasses, we examined a set of RNA-seq and DNA methylation data derived from MED12 and HMGA2 type UFs available from our previous study (5). UPA exerts its pharmacological effect by modulating progesterone receptor signaling and thereby affects proliferation, apoptosis and extracellular matrix composition (20–22). We found progesterone receptor (PGR) to be significantly upregulated in MED12 (fold change 0.38) but not in HMGA2 UFs as compared to normal myometrium (two-sided adjusted *P* = 0.009 and 0.86, respectively) (Fig. 3A). This difference was replicated in an independent set of samples (Supplementary Material, Fig. S4). Of note, non-MH UFs displayed PGR expression with levels predominantly in-between MED12 and HMGA2 UFs (Fig. 3A).

Binding sites of PGR as evaluated by ChIP-seq (23) were less methylated in MED12 UFs than in normal myometrium (adjusted *P* = 3.33 × 10^−15^, 95% CI of difference 5.2–8.2% points) or in HMGA2 tumors (adjusted *P* = 0.032, 95% CI of difference 0.39–3.8% points) (Fig. 3B), suggesting subclass specific differences in PGR activity. Compatible with these data, a principal component analysis (PCA) of 48 progesterone pathway related genes (gene ontologies GO:0050847 and GO:0032570) showed a clear separation between MED12 and HMGA2 UFs (Fig. 3C). Analysis of the expression of 84 genes involved in extracellular matrix remodeling (24) also revealed distinct expression signatures (Fig. 3D). In contrast, apoptotic signaling-pathway-related genes (24) showed no clear separation between the subclasses (Fig. 3E).

## Discussion

Recent studies have shown the existence of distinct UF subclasses (4,5). While these subclasses are associated with certain clinicopathological features, their true clinical significance has been unclear. The results from our study show for the first time that genetic UF subclass influences drug treatment response. Our data also reveal differences in progesterone receptor signaling in MED12 as compared to HMGA2 tumors, possibly underlying the observations.

The progesterone receptor modulator UPA has been the most commonly used UF drug in both Sweden and Finland, until its use was recently restricted by the European Medicines Agency to only UFs in premenopausal women for whom surgical procedures are not appropriate or have not worked, due to rare cases of liver injury. Other progesterone receptor modulators are currently under investigation for treatment of UFs. Response to UPA varies greatly between patients and individual fibroids of the same patient, suggesting that intrinsic UF features may determine the response (15,16). The reasons for this variability have been the focus of a number of recent studies. In some studies, smaller UFs were reported to be more likely to respond to treatment, others suggested better response in women with larger or fewer UFs (15,16,25). Fibroid location might also impact outcome (15,16). None of these studies considered different genetic subclasses of UF.

In our study, MED12 UFs were found to be 4.4 times more likely to shrink in response to UPA as compared to HMGA2 UFs, and this effect was largely attributed to tumors with very good response (4.7-fold). We found no association between the previously suggested predictors, initial tumor size and total number of tumors, and UPA response. Nor did women’s age, parity or sample collection site (Sweden/Finland) associate with response. Non-MH UFs are a heterogeneous group of fibroids driven by one of the more rare UF mutations or unknown changes (5). For the current study, we did not have sufficient numbers to study the more rare UF subclasses. While the non-MH UFs appeared to display an intermediate treatment response dissecting the basis of this finding would have required a much larger study.

To better understand why MED12 UFs are more sensitive to UPA as compared to HMGA2 UFs, we utilized a set of RNA-seq and DNA methylation data derived from MED12 and HMGA2 type UFs available from our previous study (5) and searched for differences in processes that have been suggested to be affected by UPA, namely progesterone signaling, extracellular matrix remodeling and apoptosis. The data revealed prominent subclass specific differences in the expression of progesterone pathway genes. The progesterone receptor gene itself, encoding the presumed target of UPA, was more highly expressed in MED12 than in HMGA2 and non-MH UFs. Compatible with active PGR signaling, PGR binding sites were hypomethylated in genomes of MED12 UFs. In PCA of gene expression data, progesterone signaling pathway genes powerfully separated MED12 and HMGA2 UFs. Thus, all layers of data examined supported a difference in PGR activity in MED12 versus HMGA2 tumors, providing a plausible explanation for the observed difference in UPA response. Compatible with our findings, it was recently demonstrated that mutation in MED12 can result in increased interaction with PGR (26). MED12 and HMGA2 UFs were also strikingly different in their expression of ECM-related genes, but not of genes related to apoptotic signaling.

While the material was uniquely suitable to address the research question at hand, the study had limitations. This was a retrospective, observational study, relying on patient records. To estimate changes in UF volume, we assessed ultrasound measurements recording the largest diameter before and after treatment as most patient records lacked more detailed volume information, or if not available relied on comments on shrinkage effect when these could be unambiguously interpreted. The UFs were measured by multiple operators using mainly ultrasonography, and the exact timing of measurement in relation to treatment varied. The potential impact of UF location could not be explored. The fibroids that responded well to the UPA treatment are likely to be underrepresented in our study material, as some may no longer have required surgery. The frequency of MED12 UFs in the Swedish as compared to the Finnish cohort was lower. All Finnish UF samples were isolated during hysterectomy while the Swedish cohort includes fibroids removed both by myomectomy and hysterectomy. Whereas most Swedish patient records contained reliable size information of only the largest fibroid(s), the Finnish records generally had size measurements from the majority of fibroids, and therefore more often allowed inclusion of more than one fibroid per patient. Small tumors were thus slightly underrepresented in the Swedish sample set. While these challenges existed they cannot explain the observed findings, subclass status emerging as an independent prognostic factor for treatment response in both arms of the current study (Swedish and Finnish). Of note, the statistical findings were in line with the presented results when effect of subclass on treatment response using only one, randomly selected UF per patient was studied (data not shown).

Tumor shrinkage has traditionally been used to measure the effect of both UPA and GnRH treatment and was therefore chosen as the readout of treatment response in this study. UPA treatment could also induce other changes than volume reduction that can result in a reduction of UF-related problems. These changes include hydropic degeneration (19), infarct-type necrosis and apoplectic changes (27). We found that, compatible with a previous report, edematous changes seem to be more common in HMGA2 tumors than MED12 tumors (19), and this feature was more frequent in HMGA2 tumors after UPA treatment. The number of samples per category was low and the statistical power of the analysis limited, but the possibility that HMGA2 tumors might respond to UPA treatment in a manner that is mechanistically different from MED12 ULs cannot be excluded.

The immediate clinical implementation of our results faces two challenges. First, it would not be straightforward to determine the UF driver mutation before treatment. Biopsy remains an option but is invasive, and use of liquid biopsy, though perhaps useful and should be examined, is not at present a proven approach in this context. Second, a person might carry multiple lesions of different subclasses, complicating matters. Our data do readily provide a potential reason for poor UPA treatment response, and might thus be useful when continuation of treatment in such cases is pondered. In moderate size submucous UFs, hysteroscopic resection of the tumor is a common treatment option, particularly in younger women with a desire to maintain fertility. Such a procedure is not always radical and complementary drug treatment may be necessary. In these cases, the genetic UF subclass could already currently be conveniently determine before treatment decisions.

The work has fundamental implications for research towards curative personalized UF management strategies. Treating UF as one disease entity in such efforts should cease. Any current and future effort should take into account the differing properties of the genetic UF subclasses.

To conclude, our data demonstrate that genetic drivers and the consequent molecular subclasses of UF predict response to UPA, with MED12 UFs showing almost five times higher probability of shrinkage as compared to HMGA2 UFs. Although the results should be validated in independent studies and the underlying mechanisms need further investigation, our finding highlights the immediate need to take genetic UF subclasses into consideration when developing and evaluating current and novel management strategies. Molecular stratification has been the norm for years in management of many forms of neoplasia, and UF patients deserve nothing less.

## Materials and Methods

The study was approved by the Finnish National Supervisory Authority for Welfare and Health (THL/151/5.05.00/2017, THL/723/5.05.00/2018), the Ethics Committee of the Hospital District of Helsinki and Uusimaa (HUS/2509/2016), and the Swedish Ethical Review Authority (2016/37-31/1 and 2016/2385-31/4).

### Study material

We retrospectively reviewed the medical records of 1657 women diagnosed with symptomatic uterine fibroids who underwent fibroid surgery at hospitals in Sweden and Finland and had given an appropriate informed consent for UF research. Our aim was to systematically include all samples informative for the research question. We identified 163 patients who underwent treatment with ulipristal acetate (UPA) before surgery, and sufficient information on treatment response could be derived for 101 tumors from 81 patients (Supplementary Material, Table S1). The reasons for excluding specimens included too short (<3 months) or unclear length of UPA treatment, or lack of measurement before and after treatment. Patients with multiple UFs where the documented size measurements and treatment response could not with certainty be linked to a particular surgically removed UF were also not included. Fibroids that did not reduce in size but showed reduced pressure symptoms, or where the pathology report described tissue softening, hyalinization or apoptotic cells were considered ‘undecided’ and were excluded from the study. Samples for which the medical record stated ‘difficult to determine shrinkage’ or ‘maybe slight shrinkage’ were also excluded. The excluded samples are described in Supplementary Material, Table S4.

The Swedish sample set consists of samples from women who underwent fibroid surgery at Danderyd Hospital between 2014 and 2019. Samples were either prospectively collected after written informed consent to participate in the study or retrieved through Stockholm Medical Biobank from women who had consented that their samples could be used for research (BbK-01616). The Finnish sample set consists of two prospective sample series (My6000 and My8000), collected between 2014 and 2019 after written informed consent. All Finnish fibroid samples were isolated during hysterectomy whereas the Swedish sample set included fibroids removed both by myomectomy and hysterectomy. Whereas most Swedish patient records contained reliable size information of only the largest fibroid(s), the Finnish records generally had size measurements from the majority of fibroids, and therefore more often allowed inclusion of several fibroids per patient. Small tumors were thus slightly underrepresented in the Swedish sample set.

All data were pseudonymized before entering the study database. Clinical data including age of the patient, duration of UPA treatment, size of the fibroids before and after treatment as assessed using ultrasound or magnetic resonance imaging (MRI), effect on bleeding, fibroid location, parity, type (hysterectomy or myomectomy) and date of surgery were retrieved from medical records. Only women with robust fibroid size measurements before and after UPA treatment or the UPA effect on fibroid size clearly documented in their medical records were included. All subjects except two had received UPA treatment (5 mg/day) for at least 3 months, and many had been treated with several 3-months courses. Two cases, one where the treatment was stopped after 2 months due to continuing tumor growth and one with clear shrinkage already after two months of treatment, were also included. Inclusion of all cases was blind to the subclass status (MED12 or HMGA2).

Histopathological evaluation of necrosis, edema and hemorrhage was conducted for all the UPA treated Finnish samples of which an H&E section was available (Supplementary Material, Table S3). For three samples, H&E sections were not available. The reactive changes were scored on a scale from −(none) to +++ (extensive). Additionally, a complementary set of untreated tumors with H&E sections was evaluated to determine a baseline for edema positivity in HMGA2 and MED12 tumors.

### Mutation analysis

#### MED12

All UF tissue samples were screened for *MED12* mutations. Genomic DNA was extracted from UF tissue samples with FastDNA Kit (MP Biomedicals LLC, Solon, OH, USA) (fresh frozen), QIAamp DNA FFPE Tissue Kit (Qiagen, Hilden, Germany) or Quick-DNA FFPE Miniprep (Zymo research, Irvine, CA, USA) (paraffin embedded archival samples) and screened for the UL causing *MED12* exon 1 and 2 mutations by Sanger sequencing. The following 5′ to 3′ primers were used on fresh frozen tissue samples CCTCCGGAACGTTTCATAGAT (*MED12* Ex1 forward) and TTCGGGACTTTTGCTCTCAC (*MED12* Ex1 reverse), and GCCCTTTCACCTTGTTCCTT (*MED12* Ex2 forward) and TGTCCCTATAAGTCTTCCCAACC (*MED12* Ex2 reverse) and for paraffin embedded archival samples: CCCCTTTTCGGCTCCCTC (*MED12* Ex1 forward) and GTCAGTGCCTCCTCCTAGG (*MED12* Ex1 reverse), and GCCCTTTCACCTTGTTCCTT (*MED12* Ex2 forward) and AAGCTGACGTTCTTGGCACT (*MED12* Ex2 reverse). *MED12* NM_005120.2 was used as a reference sequence.

PCR products were sequenced using BigDye Terminator v.3.1 sequencing chemistry (Applied Biosystems, Foster City, CA, USA) on an ABI3730 Automatic DNA Sequencer or sent for sequencing to Eurofins Genomics Germany GmbH (Ebersberg, Germany). Sequence chromatograms were analyzed manually and with Mutation Surveyor software (Softgenetics, State College, PA, USA).

#### HMGA2

The relative *HMGA2* expression was determined in all *MED12* mutation-negative samples. Total RNA was isolated from fresh-frozen UFs using TRIzol (Thermo Fisher Scientific, Waltham, MA, USA) and purified with the RNeasy MinElute Cleanup Kit (Qiagen, Hilden, Germany). Extracted RNA was converted to cDNA according to standard procedures and *HMGA2* qPCR performed on an AB 7500 Fast Real-Time PCR System with TaqMan Assay No. Hs04397751_m1 (Applied Biosystems Darmstadt, Germany), using 18S rRNA (Hs03928990_g1) as endogenous control. Analyses were performed as triplicates and all runs contained a previously assessed UL sample showing weak *HMGA2* expression (negative control) and another showing a relative *HMGA2* expression >100 (positive control) when compared to the negative control.

Paraffin embedded archival UF samples were screened for *HMGA2* expression using an anti-HMGA2 antibody (1:2000, 59170AP, Biocheck Inc., Foster City, CA, USA). Heat-induced antigen retrieval was followed by endogenous peroxidase blocking and incubation with primary antibody overnight at +4°C. The immunohistochemical staining was visualized by Vectastain Elite ABC HRP kit (Vector Laboratories Burlingame, CA, USA) and DAB substrate kit (Fisher Scientific, Waltham MA, USA). Each set of staining included a positive control. Myometrium was used as a negative control. The intensity of the immunoreaction was classified into four groups: −= fully negative, (+) = single cell positivity, + = low positivity, ++ = strong positivity. Only samples that showed strong HMGA2 positivity were considered overexpressed.

### Gene expression data

Existing RNA-sequencing (RNA-seq) data from 42 MED12 and 44 HMGA2 UFs as previously described (5) were used for differential expression analyses and principal component analyses (PCA). Differential expression analysis was performed with DESeq2 (28), including the sequencing batch as a confounder, and reported as two-sided, Benjamini-Hochberg adjusted (FDR) *P*-values (Wald test) and base-2 log fold-changes (logFC). For visualization and PCA of gene expression values, the effect of the sequencing batch was removed with limma (v. 3.42.0) removeBatchEffect after variance stabilizing transformation (DESeq2) for log-scaled estimates of gene expression.

A complementary set of expression array material was used to replicate our observation of *PGR* expression level differences in an independent set of tumors. The expression array material (Affymetrix GeneChip Human Exon 1.0 ST) was described previously (29). We analyzed normal myometrium (*n* = 44) samples together with an independent replication set of HMGA2 (*n* = 17) and MED12 (*n* = 32) tumors after excluding any samples overlapping the RNA-seq material above. Analysis of gene expression data was performed with Partek Genomic Suite version 6.5, including quantile-normalization by the robust multichip average method and batch effect removal as described previously (29). Two-tailed *t*-test was used to test for difference of the resulting group means.

### ChIP-seq and methylation

Progesterone receptor ChIP-seq reads were downloaded from GEO dataset GSE138049. Raw sequencing reads were quality and adapter trimmed with cutadapt version 1.16 in Trim Galore version 0.3.7 using default parameters. Trimmed reads were aligned to the hs37d5 reference genome using Bowtie 2 (version 2.1.0) and reads with mapping quality<20 were filtered out with samtools (version 1.7). Peak calling for reads with mapping quality>20 was performed with MACS2 (version 2.1.2) with default parameters except FDR cutoff for narrowPeak regions was set to 0.01. One sample (PT1) was excluded due to low amount of narrowPeaks (*n* = 34) and low fraction of reads in peaks (FRiP, 0%). Other samples had from 7776 to 48 734 narrowPeaks and at least 1% FRiP. The ENCODE blacklist genomic regions (30) were filtered out from the final narrowPeaks located at autosomes and X chromosome. Peak overlap analysis of PGR ChIP-seq samples were performed with DiffBind version 2.14.0 in R 3.6.3 (31). Duplicate reads were removed with samtools (version 1.7) rmdup from the ChIP alignment files that were utilized in the DiffBind analysis. Counting of reads in the peaks was performed by extending reads to average fragment length that were derived from MACS. All narrowPeaks that overlap in at least three samples (dba.count-function with parameter minOverlap = 3) were included in the peakset, and these consensus peaks were re-centered around a consensus summit including 250 bp up- and downstream of the summit. The resulting peakset consisted of 13 385 progesterone receptor binding sites.

Existing Nanopore-sequencing data from 96 normal myometrium, 28 HMGA2 ULs, 13 MED12 ULs and 61 ULs wild type for HMGA2 and MED12 (5) were used for CpG methylation analyses. Methylation status for each read at each CpG site aligned to the reference genome was called with Nanopolish (32) as described earlier (5). Mean genome-wide methylation was computed on CpG sites with 2–60× coverage as the sum of methylated calls out of all calls. The average methylation for each sample over ChIP-seq peak summits was calculated as mean (over all sites +-250 bp distance from region center) of mean (for each distance from region center over all regions in the peakset) of mean (for a specific CpG over the reads covering that site) methylation. The statistical significance for the overall methylation difference over an annotation set was assessed with ordinary least squares regression with sample mean methylation as dependent variable, and mean genome-wide methylation and sample subclass as independent variables. The sample subclass was encoded as treatment with respect to normal samples. Pairwise differences were tested with *t*-test on contrasts in the regression model.

### Statistical methods

No statistical methods were used to predetermine sample size. The final sample size was determined by the number of available samples that met the inclusion criteria. Differences in treatment responses were assessed with logistic regression having treatment responses as the dependent variable and tumor subclasses as the independent variable. Further independent variables were included in a multivariable model to adjust for cohort (Sweden or Finland Myoma Study), patient age at surgery (years), tumor diameter at start of treatment (mm), parity (nulliparous or parous) and total number of fibroids (one or multiple); missing data were excluded from the multivariable analysis. Odds-ratios, 95% confidence intervals (95% CI) and two-sided *P*-values were calculated using generalized estimating equations (GEE; R version 4.1.1; geepack version 1.3-2): exchangeable correlation structure, binomial random component logit link function and sandwich variance estimates were used to account for any patients with multiple tumors (i.e. multiple observations per patient). Following a Bonferroni correction for two independent tests (MED12 versus HMGA2 and MED12 versus wild type), *P*-values <0.05/2 were considered significant. Post hoc analyses were carried out to better understand the weight of the ‘very good’ treatment responses and known confounding factors, such as tumor diameter.

### Data availability

Genetic data presented in this manuscript have been deposited at the European Genome-phenome Archive (EGA) under accession number EGAS00001004499. A data access committee (DAC) has been established by two University of Helsinki representatives that are independent of the authors of the current study. Requests for the data should be sent to the DAC via email (dac-finlandmyomastudy@helsinki.fi).

The DAC ensures that the intended use of data as detailed in the request is compatible with the requirements of the European General Data Protection Regulation (GDPR), consistent with the consents given and otherwise ensures the protection of data subjects’ rights as required by the GDPR. The DAC will always grant access to the data if the University is legally allowed to do so without infringing the rights and freedoms of data subjects. Subject to the requirements of the GDPR, the DAC grants access to the genetic data to non-commercial academic research on neoplasia and chromatin.

PGR ChIP-seq data were downloaded from GEO database, accession code GSE138050. For RNA-seq, the reference annotation for GRCh37 was downloaded from Ensembl (ftp://ftp.ensembl.org/pub/release-75/gtf/homo_sapiens/).

### Code availability

No custom code was utilized in this work. The packages and software used are described in the Methods section.

## Supplementary Material

HMG-2022-CE-00190_Kolterud_Table_S1_ddac217

HMG-2022-CE-00190_Kolterud_Table_S3_Histopathological_evaluation_ddac217

HMG-2022-CE-00190_Kolterud_Table_S4_Clinical_characteristics_ddac217

HMG-2022-CE-00190_R2_Kolterud_Supplementary_Appendix_ddac217
