# Supplementary material for: Molecular subclass of uterine fibroids predicts tumor shrinkage in response to ulipristal acetate
Source: Hum Mol Genet. 2022 Sep 1;32(7):1063–71. doi: 10.1093/hmg/ddac217 (PMC10026225; doi:10.1093/hmg/ddac217)
Supplement: HMG-2022-CE-00190_R2_Kolterud_Supplementary_Appendix_ddac217 [file hmg-2022-ce-00190_r2_kolterud_supplementary_appendix_ddac217.docx]

**Supplementary Appendix**

Supplement to: Molecular Subclass of Uterine Fibroids Predicts Tumor Shrinkage in Response to Ulipristal Acetate

Åsa Kolterud, Niko Välimäki, Heli Kuisma, Joonatan Patomo, Sini T. Ilves, Netta Mäkinen, Jaana Kaukomaa, Kimmo Palin, Eevi Kaasinen, Auli Karhu, Annukka Pasanen, Ralf Bützow, Oskari Heikinheimo, Helena Kopp Kallner & Lauri A. Aaltonen

# **Table of Contents**

**Supplementary Figures and Tables** 2

Figure S1 Tumor diameter at start of UPA treatment. 2

Figure S2 Change in tumor diameter from start to end of UPA treatment. 3

Figure S3 Resulting simple and multivariable logistic regression models. 4

Figure S4 *PGR* expression difference in an independent set of tumor samples. 5

Table S2 Overview of the patient and tumor material. 6

# **Supplementary Figures and Tables**


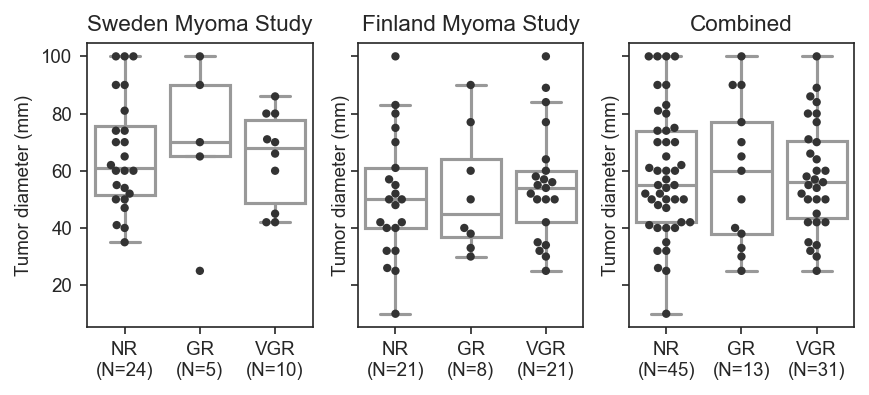


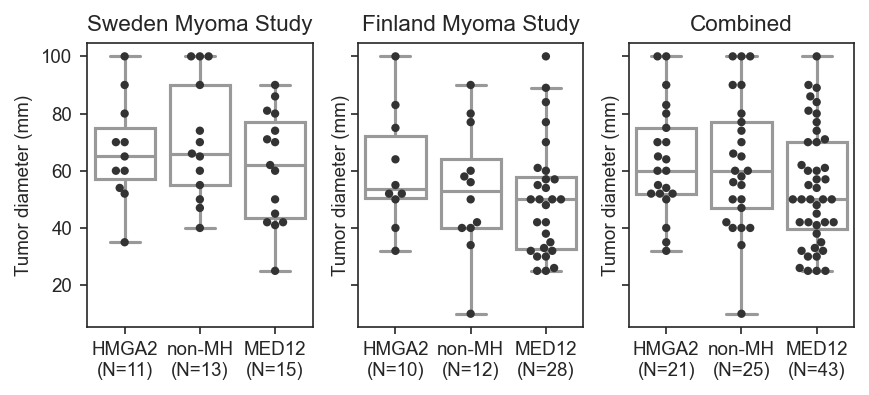


## Figure S1 Tumor diameter at start of UPA treatment.

On the top, tumor diameter (mm) stratified by treatment response, and on the bottom, tumor diameter (mm) stratified by subclass. Altogether 89 tumors had this information available. Overall median values were 65mm (IQR 51-80mm) and 50mm (IQR 39-61mm) for the Sweden and Finland Myoma Study material, respectively. Boxplots show the median and the first and third quartiles; whiskers extend up to 1.5 IQR beyond the quartiles. VGR: very good response to treatment; GR: good response; NR: non-responding; MED12: *MED12* mutation positive tumors; HMGA2: *HMGA2* overexpressing tumor; non-MH: wild-type for *MED12* and *HMGA2*.


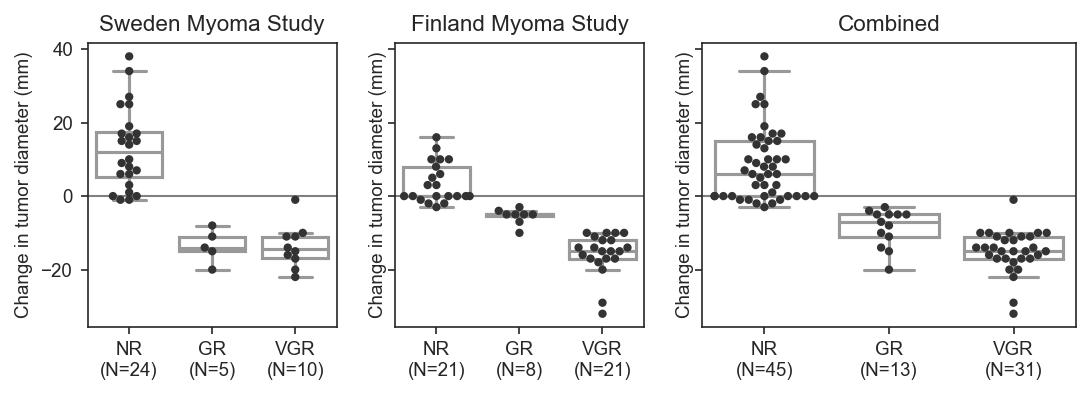


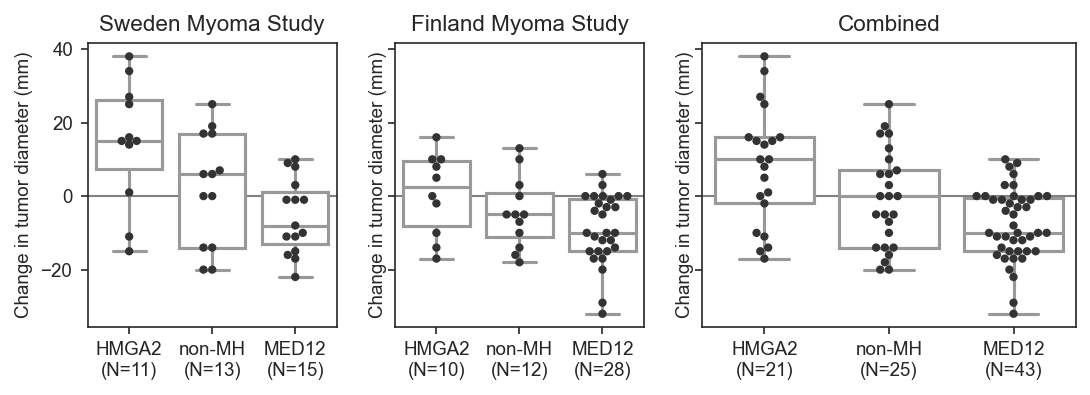


## Figure S2 Change in tumor diameter from start to end of UPA treatment.

On the top, change in tumor diameter (mm) stratified by treatment response, and on the bottom, change in tumor diameter (mm) stratified by subclass. Y-axis shows the difference in the longest diameter before and after UPA treatment: all Y-axes have the same scale, and negative values correspond to decreased diameter after treatment. Altogether 89 tumors had this information available. Boxplots show the median and the first and third quartiles; whiskers extend up to 1.5 IQR beyond the quartiles. VGR: very good response to treatment; GR: good response; NR: non-responding; MED12: *MED12* mutation positive tumors; HMGA2: *HMGA2* overexpressing tumor; non-MH: wild-type for *MED12* and *HMGA2*.


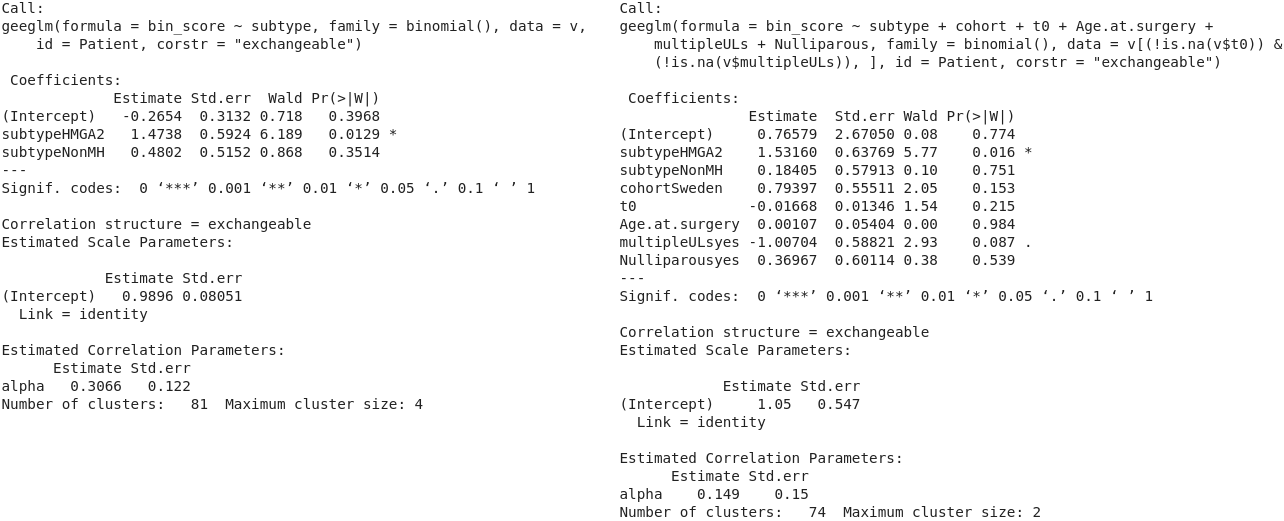


## Figure S3 Resulting simple and multivariable logistic regression models.

On the left and right, simple and multivariable logistic regression models, respectively. Estimates are in log-scale. Treatment responses (VGR/GR versus NR) were used as the dependent variable and tumor subclasses (MED12, HMGA2 or non-MH) as the independent variable. In the multivariable model, cohort information was encoded as either Finland or Sweden; tumor diameter unit was in millimeters; age was in years; number of tumors per patient was either single or multiple UFs; and parity was either nulliparous or parous. The multivariable model excluded patients with missing data (n=74 patients remained).


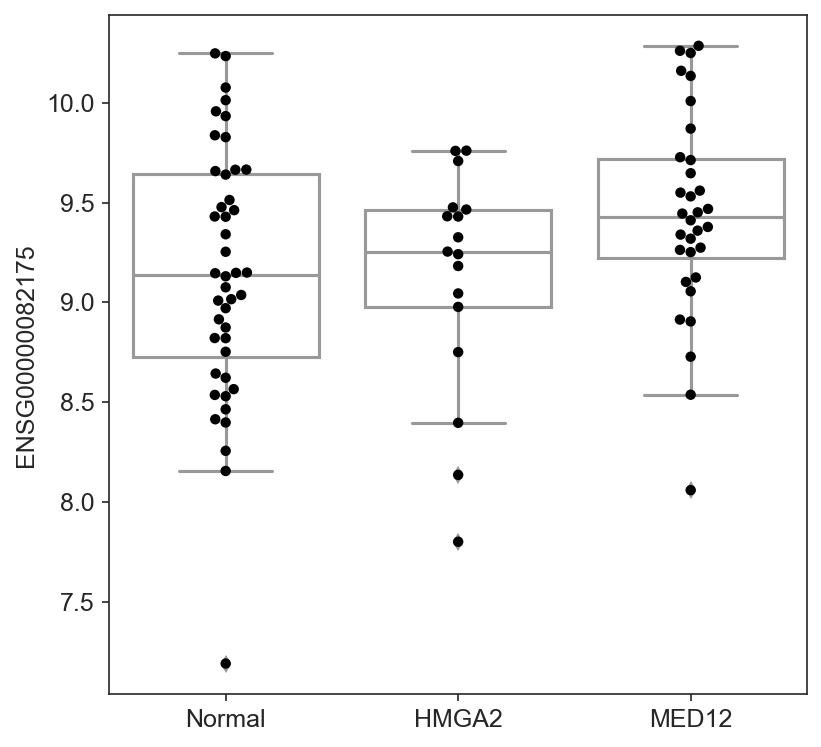


## Figure S4 *PGR* expression difference in an independent set of tumor samples.

*PGR* expression analysis in an independent set of tumor samples, which were available from a previously published expression array study (29). The material here provides an independent replication of the *PGR* gene expression differences observed in **Figure 3**. Y-axis shows quantile-normalized, robust multichip average estimates of *PGR* gene expression. From left to right, *PGR* expression in normal myometrium (n=44), and in HMGA2 (n=17) and MED12 (n=32) tumors. Box plots show the median and the first and third quartiles; whiskers extend up to 1.5 IQR. Dots show individual data points. The difference between HMGA2 and normal myometrium in *PGR* expression was smaller (difference of means=-0.02; two-tailed t-test P=0.922) than the difference observed between MED12 and normal myometrium (difference of means=0.297; P=0.032).

## Table S2 Overview of the patient and tumor material.

|  | **Sweden Myoma Study** | **Finland Myoma Study** |
| --- | --- | --- |
| Number of patients | 40 | 41 |
| Duration of UPA treatment (months) | 4 (3-6) | 3 (3-6) |
| Age at surgery (years) | 45 (42-49) | 46 (44-49) |
| Operation |  |  |
| Hysterectomy | 31 (77.5%) | 41 (100.0%) |
| Myomectomy | 9 (22.5%) | 0 (0.0%) |
| Nulliparous | 9 (22.5%) | 13 (31.7%) |
| Total number of UFs per patient | 2 (1-3) | 2 (1-3) |
| Number of UFs in this study | 45 | 56 |
| UF diameter at start (mm) | 65 (51-80) | 50 (39-61) |
| Location |  |  |
| Intramural | 24 (66.7%) | 31 (75.6%) |
| Submucous | 7 (19.4%) | 8 (19.5%) |
| Subserous | 5 (13.9%) | 2 (4.9%) |

Data shown either as a median and interquartile range, or as a number and percentage. Location information was available for 77 tumors.
